# Chronic pulmonary exposure to traffic-related fine particulate matter causes brain impairment in adult rats

**DOI:** 10.1186/s12989-018-0281-1

**Published:** 2018-11-09

**Authors:** Chi-Hsiang Shih, Jen-Kun Chen, Li-Wei Kuo, Kuan-Hung Cho, Ta-Chih Hsiao, Zhe-Wei Lin, Yi-Syuan Lin, Jiunn-Horng Kang, Yu-Chun Lo, Kai-Jen Chuang, Tsun-Jen Cheng, Hsiao-Chi Chuang

**Affiliations:** 1 School of Respiratory Therapy, College of Medicine, Taipei Medical University, Taipei, Taiwan; 2 Institute of Biomedical Engineering & Nanomedicine, National Health Research Institutes, Miaoli, Taiwan; 3 Graduate Institute of Environmental Engineering, National Taiwan University, Taipei, Taiwan; 4 Department of Physical Medicine and Rehabilitation, Taipei Medical University Hospital, Taipei, Taiwan; 5 Department of Physical Medicine and Rehabilitation, School of Medicine, College of Medicine, Taipei Medical University, Taipei, Taiwan; 6 The Ph.D Program for Neural Regenerative Medicine, College of Medical Science and Technology, Taipei Medical University, Taipei, Taiwan; 7 School of Public Health, College of Public Health, Taipei Medical University, Taipei, Taiwan; 8 Department of Public Health, School of Medicine, College of Medicine, Taipei Medical University, Taipei, Taiwan; 9 Institute of Occupational Medicine and Industrial Hygiene, College of Public Health, National Taiwan University, Taipei, Taiwan; 10 Division of Pulmonary Medicine, Department of Internal Medicine, Shuang Ho Hospital, Taipei Medical University, New Taipei City, Taiwan

**Keywords:** Air pollution, Inflammation, Memonry deficiency, Neurotoxicity, Oxidative stress

## Abstract

**Background:**

Effects of air pollution on neurotoxicity and behavioral alterations have been reported. The objective of this study was to investigate the pathophysiology caused by particulate matter (PM) in the brain. We examined the effects of traffic-related particulate matter with an aerodynamic diameter of < 1 μm (PM_1_), high-efficiency particulate air (HEPA)-filtered air, and clean air on the brain structure, behavioral changes, brainwaves, and bioreactivity of the brain (cortex, cerebellum, and hippocampus), olfactory bulb, and serum after 3 and 6 months of whole-body exposure in 6-month-old Sprague Dawley rats.

**Results:**

The rats were exposed to 16.3 ± 8.2 (4.7~ 68.8) μg/m^3^ of PM_1_ during the study period. An MRI analysis showed that whole-brain and hippocampal volumes increased with 3 and 6 months of PM_1_ exposure. A short-term memory deficiency occurred with 3 months of exposure to PM_1_ as determined by a novel object recognition (NOR) task, but there were no significant changes in motor functions. There were no changes in frequency bands or multiscale entropy of brainwaves. Exposure to 3 months of PM_1_ increased 8-isoporstance in the cortex, cerebellum, and hippocampus as well as hippocampal inflammation (interleukin (IL)-6), but not in the olfactory bulb. Systemic CCL11 (at 3 and 6 months) and IL-4 (at 6 months) increased after PM_1_ exposure. Light chain 3 (LC3) expression increased in the hippocampus after 6 months of exposure. Spongiosis and neuronal shrinkage were observed in the cortex, cerebellum, and hippocampus (neuronal shrinkage) after exposure to air pollution. Additionally, microabscesses were observed in the cortex after 6 months of PM_1_ exposure.

**Conclusions:**

Our study first observed cerebral edema and brain impairment in adult rats after chronic exposure to traffic-related air pollution.

**Electronic supplementary material:**

The online version of this article (10.1186/s12989-018-0281-1) contains supplementary material, which is available to authorized users.

## Background

Pulmonary exposure to air pollution was reported to cause central nervous system (CNS) toxicity and is associated with a risk of neurological diseases. For example, observations from the U.S. Department of Veterans Affairs Normative Aging Study cohort showed associations of traffic-related pollution and black carbon (BC) with cognition function [1]. They observed an association between traffic-related air pollution and deficiencies in cognitive function in older men. Exposure of high levels of coarse particulate matter (PM_2.5–10_) and fine particulate matter (PM_2.5_) was associated with faster cognitive decline as reported by the Nurses’ Health Study Cognitive Cohort in the US [2]. Furthermore, PM_2.5_exposure was associated with an increase in hospital admission in patients with dementia and Parkinson’s disease (PD) in 50 northeastern US cities [3]. Based on these epidemiological reports, pulmonary exposure to air pollution could result in the development of neurological disease. However, the effects of air pollution on neurotoxicity remain unclear.

Toxicological evidence has confirmed the epidemiological associations between neurotoxicity and particulate air pollution. Neuroinflammation is considered to be an important biological response to PM exposure. Pro-inflammatory cytokines such as tumor necrosis factor alpha (TNF-α) and interleukin-1 alpha (IL-1α) were increased in brain after PM exposure [4, 5]. Alteration in IL-6 expression was also observed in the hippocampus after PM exposure [6]. Generally, inflammatory responses in the lungs due to particulate air pollution are known to result in adverse cardiopulmonary health effects [7]; however, brain inflammation occurring due to air pollution is still poorly understood. Inflammation is defined as a non-specific protective response, which is believed to be a critical step in removing injurious stimuli and initiating the healing process. Furthermore, oxidative stress is recognized as a pathogenic mechanism of neurodegenerative diseases. The physicochemistry of PM is an important determinant in regulating PM bioreactivity. Inhaled ultrafine PM (< 100 μm) causes oxidative-inflammatory reactions at sites of deposition [8]. Therefore, exposure to PM may increase oxidative stress and cerebral vascular injury, leading to systemic and local effects in different brain regions [9].

Long-term exposure to air pollutants may cause neurological effects in susceptible groups. Also, the overall risk of neurological disease attributable to air pollution might be considerably higher than previously thought [10]. However, the pathways of PM-induced neurotoxicity remain unclear. A previous study showed that magnetite PM of < 200 nm in diameter was observed in the human brain [11], which demonstrated direct evidence of lung-to-brain effects of PM. Currently, there are three hypothesized pathways of PM’s effects on the brain. First, the olfactory bulb is considered to be one route by which inhaled PM can be directly translocated into the brain [11]. Second, smaller PM can directly cross the blood-brain barrier (BBB), which is responsible for keeping harmful particles and chemicals out of the brain [12, 13]. Third, PM directly/indirectly damages barriers, such as the BBB and nasal epithelium [14]. Repeated injury of these barriers is able to impair their integrity, leading to PM and others (e.g. neurotoxic plasma-derived components, cells, and pathogens) crossing the barriers and entering the brain. Additionally, inhaled PM associated with emphysema formation [15] and oxygen desaturation [16, 17], which could lead to hypoxia development. The impairment of oxygen and nutrient supply system may result in brain function alteration. Therefore, exploring the brain pathophysiology that occurs with exposure of PM is urgently needed. The objective of this study was to investigate the effects of particulate air pollution on the brain. Adult Sprague-Dawley (SD) rats were whole-body exposed to traffic-related PM_1_ (< 1 μm) for 3 and 6 months, followed by a magnetic resonance imaging (MRI) analysis, behavioral observations, and a brainwave analysis. The particle size distribution, PM_1_ mass concentration, particle number concentration, lung deposition surface area (LDSA) concentration, and BC mass concentration were continuously monitored during the study period. Bioreactivity was determined in brain sections (cortex, cerebellum, and hippocampus), the olfactory bulb, and serum.

## Results

### Exposure to particulate air pollution

**Fig. 1:**
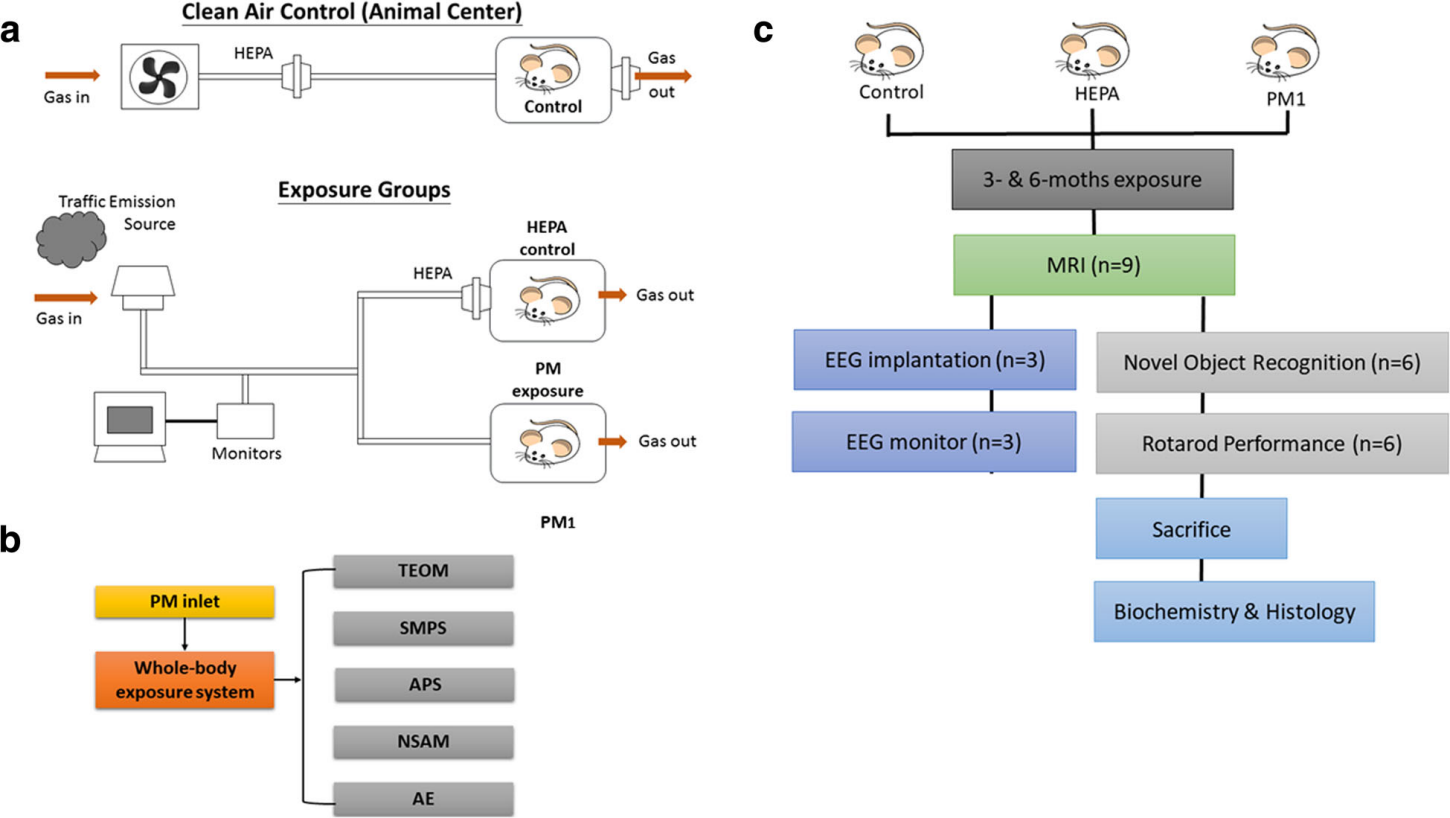
Overview of the experimental design for investigating the effects of particulate air pollution on the rat brain in vivo. **a** Illustration of the exposure systems and conditions of traffic-related air pollution by whole-body exposure in 6-month-old SD rats. Rats were randomly divided into three exposure conditions, including a clean air control group (*n* = 18) in the Laboratory Animal Center, a high-efficiency particulate air (HEPA)-filtered air control group (*n* = 18), and a particulate matter with an aerodynamic diameter of < 1 μm (PM_1_) group (*n* = 18). **b** Ambient air was sampled by an omnidirectional PM inlet located on the roof of a station and then introduced into whole-body exposure system. Particle physical characteristics were determined using a tapered element oscillating microbalance (TEOM), a scanning mobility particle sizer (SMPS), an aerodynamic particle sizer (APS), a nanoparticle surface area monitor (NSAM) and an sethalometer (AE). **c** Rats in each groups were randomly selected for a magnetic resonance imaging (MRI) analysis (*n* = 9), electroencephalography (EEG) implementation and monitoring (*n* = 3), novel object recognition (NOR) task (*n* = 6), and rotarod performance (*n* = 6) after 3 and 6 months of exposure to traffic-related air pollution

**Fig. 2:**
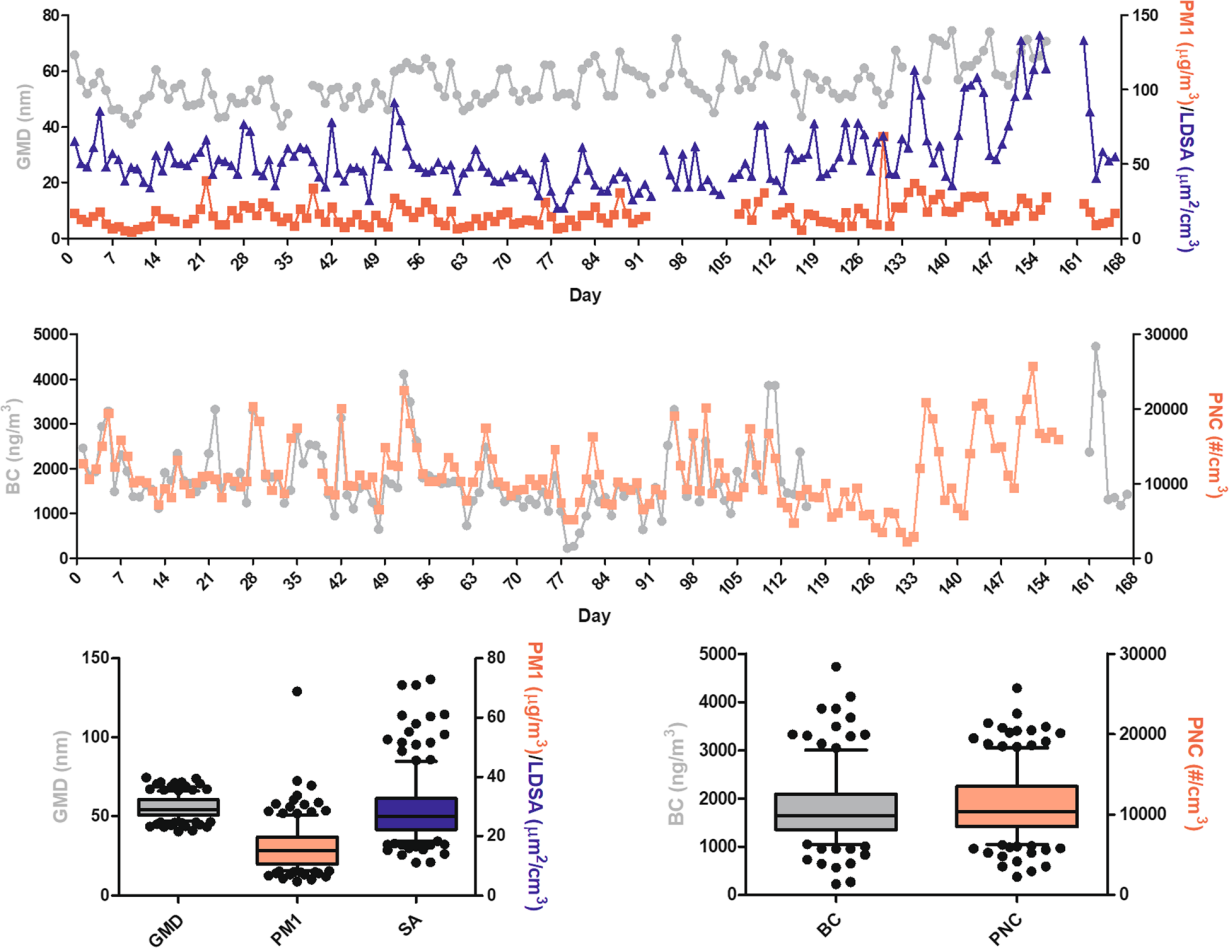
Measurement of whole-body exposure to particulate air pollution in rats during the study period. Daily distributions of the geometric mean diameter (GMD), particulate matter with an aerodynamic diameter of < 1 μm (PM_1_) mass concentration, and lung deposition surface area (LDSA) in the alveolar region concentration, black carbon (BC), and particle number concentration (PNC) during the study period were determined. Characteristics of the traffic-related particles were as follows: the GMD was 55.8 nm; the PM_1_ mass concentration was 16.3 μg/m^3^; the LDSA was 55.1 μm^2^/cm^3^; the BC was 1800 ng/m^3^; and the PNC was 11257 μg/m^3^

The exposure system and experimental design is shown in Fig. 1. Rats were continuously exposed to 24 weeks (6 months) of traffic-related PM_1_ during the study period. Daily distributions of the geometric mean diameter (GMD), PM_1_ mass concentration, LDSA in the alveolar region, BC, and particle number concentration (PNC) are shown in Fig. 2. The mean value ± SD (min~max) of the GMD over the entire observation period was 55.8 ± 7.3 (40.3~ 74.5) nm. Results showed that most of the particles to which the rats were exposed were in ultrafine size fractions (< 100 nm). We observed that the PM_1_ mass concentration was 16.3 ± 8.2 (4.7~ 68.8) μg/m^3^, and the LDSA in the alveolar region was 55.1 ± 21.7 (20.7~ 136.6) μm^2^/cm^3^. The BC mass concentration was 1800 ± 784 (219~ 4732) ng/m^3^, and the PNC was 11,257 ± 4388 (2218~ 25,733) #/m^3^.

### Brain volume

**Fig. 3:**
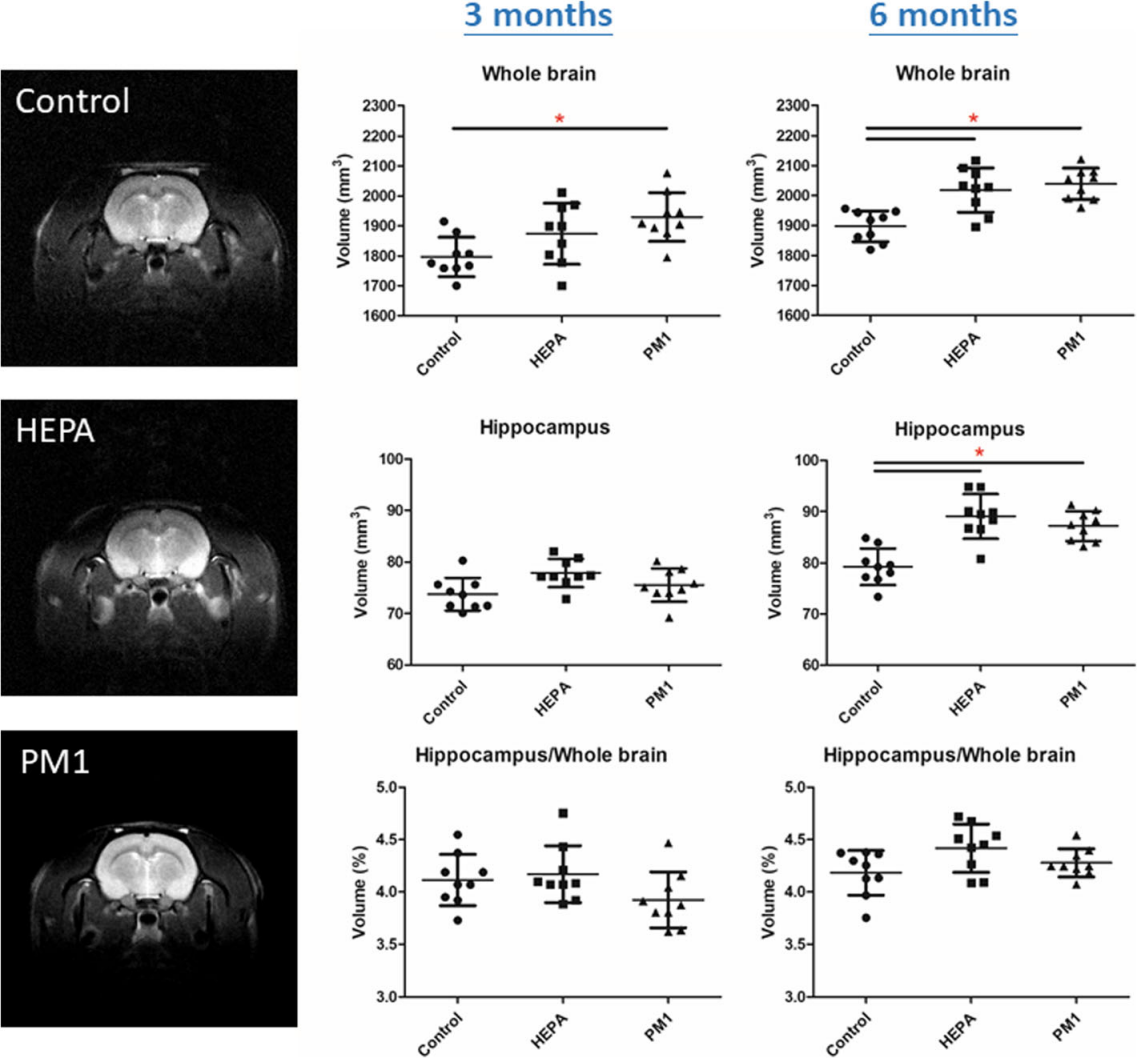
Magnetic resonance imaging (MRI) analysis of rat brains in the control, high-efficiency particulate air (HEPA), and particulate matter with an aerodynamic diameter of < 1 μm (PM_1_) groups (*n* = 9). The whole brain volume, hippocampal volume, and hippocampus to whole brain volume (%) were measured after 3 and 6 months of exposure. Whole brain volumes (nm^3^) of rats exposed to PM_1_ for 3 months and to HEPA and PM_1_ for 6 months were significantly larger than those of the control. Hippocampal brain volumes (nm^3^) of rats exposed to HEPA and PM_1_ for 6 months were significantly larger than those of the control. * *p* < 0.05

To understand the effect of chronic pulmonary exposure to traffic-related PM_1_ on the rat brain, we first examined alterations in body weight after 3 and 6 months of exposure. Body weights of rats after exposure to PM_1_ are shown in Additional file 1: Figure S1. We observed that the rats in the HEPA and PM_1_ groups had significant decreases in body weight after 3 and 6 months of exposure compared to the control group (*p* < 0.05). Next, we analyzed brain images of the control, HEPA, and PM groups by MRI. Brain MRI images were used to calculate the volumes of the whole brain and hippocampus in the control, HEPA, and PM_1_ groups as shown in Fig. 3. Although the body weight was significantly lower in rats in the HEPA and PM_1_ groups, we observed that the whole brain volume (nm^3^) of 3-month PM_1_-exposed rats was significantly larger than those of the control and HEPA rats (*p* < 0.05), but there were no significant differences in hippocampal volumes (nm^3^) among the control, HEPA, and PM groups. Notably, whole-brain and hippocampal volumes in rats of the HEPA and PM_1_ groups were significantly larger than those of control rats at 6 months of exposure (*p* < 0.05). The ratio of the hippocampal volume to whole brain volume (vol%) was next investigated (Fig. 3). We observed that the ratio of the hippocampal to total brain volume (vol%) was slightly lower in the PM_1_ group than in the control group after 3 months of exposure, but lower levels were not observed after 6 months of exposure.

### Behavior

**Fig. 4:**
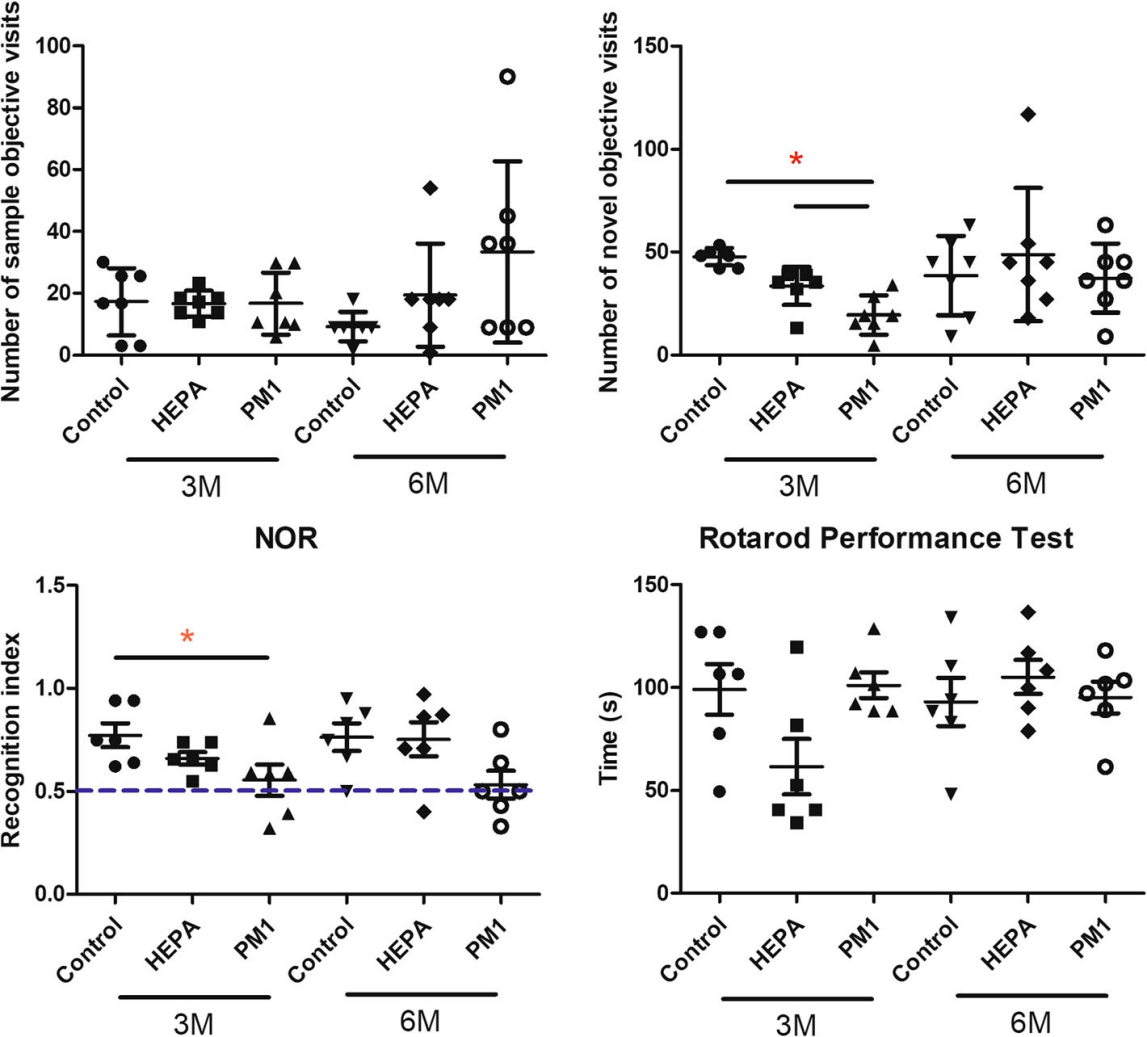
Behavioral observations in rats after 3 and 6 months of exposure to traffic-related air pollution. The novel object recognition (NOR) task and rotarod performance task (*n* = 6) were conducted in rats of the control, high-efficiency particulate air (HEPA), and particulate matter with an aerodynamic diameter of < 1 μm (PM_1_) groups. Three months of exposure to PM_1_ caused significant reductions in the number of novel object visits and recognition index compared to the control and HEPA groups. There were no differences in the rotarod performance test among the groups after exposure. * *p* < 0.05

Behavioral observations included the NOR test and rotarod performance test, and results are shown in Fig. 4. There were no significant changes in the number of sample objective visits by the control, HEPA, and PM_1_ groups after 3 and 6 months of exposure. However, we observed that rats in the PM_1_ group had significant fewer novel objective visits after 3 months of exposure compared to the control and HEPA groups (*p* < 0.05). Similarly, after 3 months of exposure to PM_1_, rats had a significantly lower recognition index compared to the control (*p* < 0.05). Also, values of the recognition index after 3 and 6 months of exposure to PM_1_ were close to the bottom levels (0.50) of recognition memory. We next examined motor function using a rotarod performance test, but there were no significant differences among the control, HEPA, and PM_1_ groups after 3 and 6 months of exposure.

### Brainwaves

**Fig. 5:**
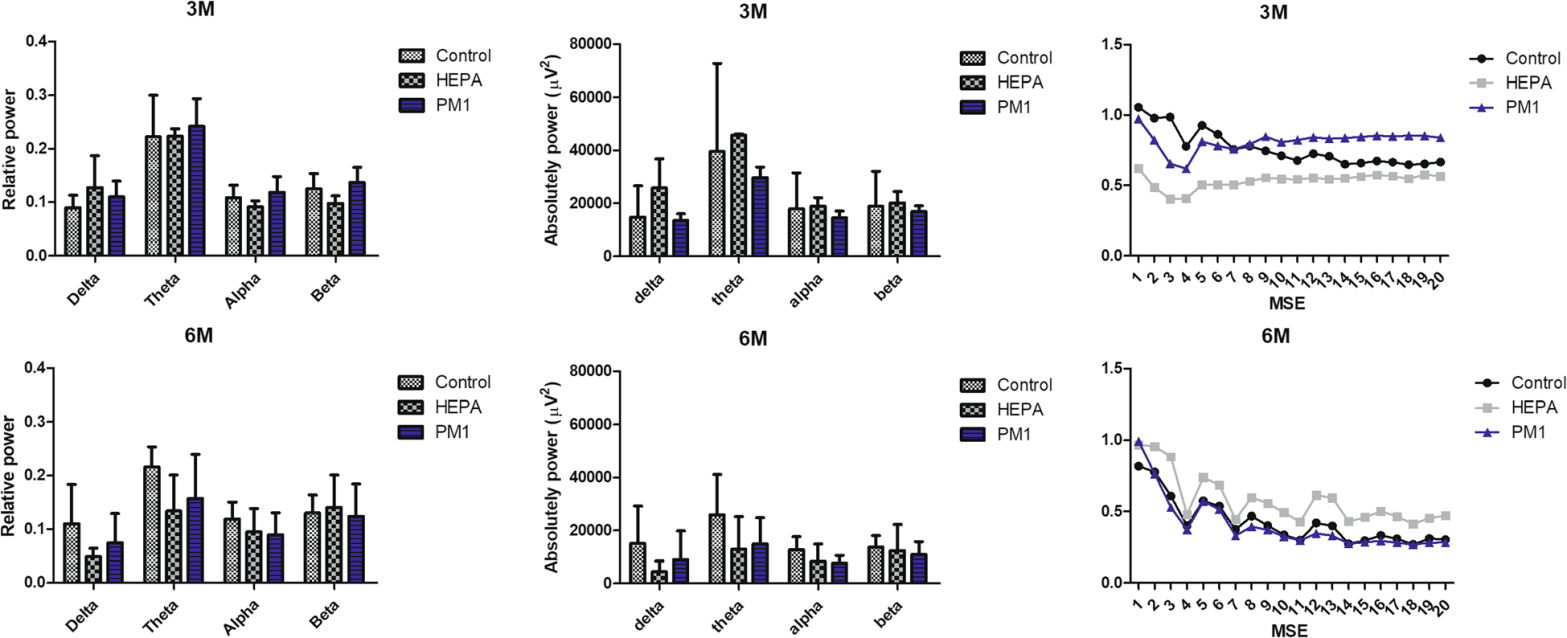
Electroencephalography (EEG) monitoring in rats after 3 and 6 months of exposure to traffic-related air pollution. The relative power, absolutely power, and multiscale entropy (MSE), of rats in the control, high-efficiency particulate air (HEPA), and particulate matter with an aerodynamic diameter of < 1 μm (PM_1_) after 3 and 6 months of exposure were calculated. There were no significant differences in the four frequency bands (delta, theta, alpha, and beta) of relative power, absolutely power, or MSE among the control, HEPA, and PM_1_ groups after 3 and 6 months of exposure

EEG characteristics were analyzed as shown in Fig. 5. The relative power and absolutely power of rats in the control, HEPA, and PM_1_ groups after 3 and 6 months of exposure were calculated. There were no significant differences in the four frequency bands (delta, theta, alpha, and beta) of the relative power and absolute power among the control, HEPA, and PM_1_ groups after 3 and 6 months of exposure. We further examined the MSE in rats, which showed no significant differences among the control, HEPA, and PM_1_ groups after 3 and 6 months of exposure.

### Biochemistry

**Fig. 6:**
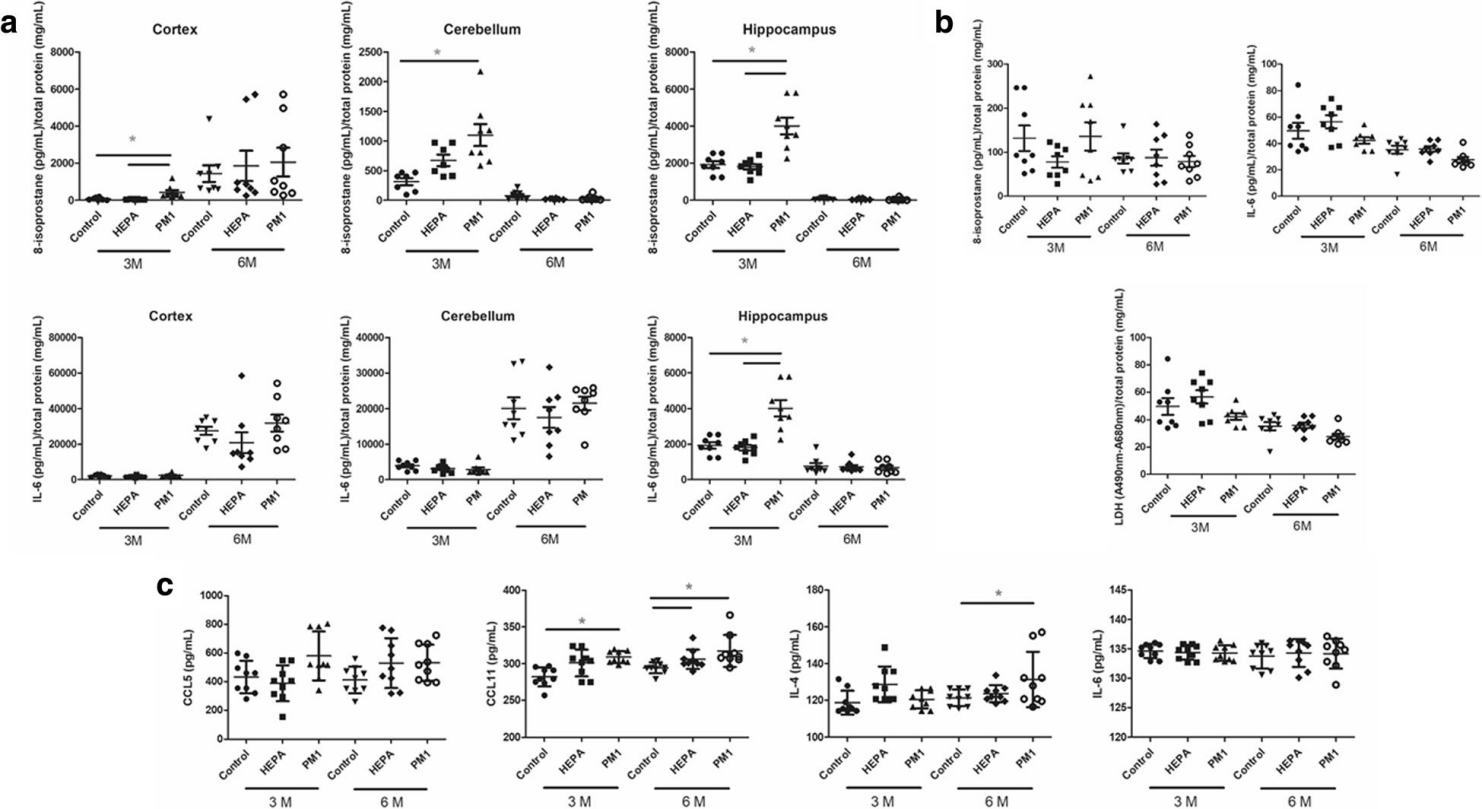
Bioreactivity of the brain, olfactory bulb, and serum of rats after 3 and 6 months of exposure to traffic-related air pollution. **a** 8-Isoprostane and interleukin (IL)-6 levels were determined in the cortex, cerebellum, and hippocampus in rats of the control, high-efficiency particulate air (HEPA), and particulate matter with an aerodynamic diameter of < 1 μm (PM_1_). Levels of 8-isoprostane in the cortex, cerebellum, and hippocampus of rats significantly increased after 3 months of exposure. Levels of IL-6 in the hippocampus significantly increased after 3 months of exposure. **b** 8-Isoprostane, IL-6 and lactate dehydrogenase (LDH) levels were determined in the olfactory bulb of rats in the control, HEPA, and PM_1_ groups. There were no significant differences among groups after exposure. **c** CCL5, CCL11, IL-4, and IL-6 were determined in the serum of rats in the control, HEPA, and PM_1_ groups. Levels of CCL11 had significantly increased after 3 months of exposure to PM_1_ and after 6 months of exposure to PM_1_ and HEPA. IL-4 had significantly increased after 6 months of exposure to PM_1_. * *p* < 0.05

Figure 6 shows biological effects on the brain, olfactory bulb, and serum of rats in the control, HEPA, and PM_1_ groups after 3 and 6 months of exposure. First, three sections of the brain were collected, including the cortex, cerebellum, and hippocampus, for 8-isoprostane and IL-6 determinations. We observed that PM_1_ exposure significantly increased levels of 8-isoprostane in the cortex (*p* < 0.05; compared to the control and HEPA groups), cerebellum (*p* < 0.05; compared to the control group), and hippocampus (*p* < 0.05; compared to the control and HEPA groups) after 3 months of exposure (Fig. 6a). However, differences in the three brain sections were not observed after 6 months of exposure. We observed that IL-6 levels in the hippocampus were significantly increased by 3 months of exposure to PM_1_ compared to the control and HEPA groups (*p* < 0.05; Fig. 6a), but a similar situation was not observed in the cortex or cerebellum. Similar to 8-isoprostane, we did not observe significant differences in IL-6 in the cortex, cerebellum, and hippocampus after 6 months of exposure. Next, we observed 8-isoprostane, IL-6 and LDH levels in the olfactory bulb of rats of the control, HEPA, and PM_1_ groups after 3 and 6 months of exposure (Fig. 6b). There were no significant alterations in 8-isoprostane, IL-6, or LDH levels in the olfactory bulb in rats among the groups after exposure. We then examined levels of CCL5, CCL11, IL-4, and IL-6 in serum among the three groups after 3 and 6 months of exposure (Fig. 6c). Serum levels of CCL11 were significantly high after 3 and 6 months of exposure to PM_1_ compared to control rats (*p* < 0.05), whereas CCL11 was significantly higher after 6 months of exposure to HEPA air compared to control rats (*p* < 0.05). We also observed that serum IL-4 was significantly higher after 6 months of exposure to PM_1_ compared to controls rats (*p* < 0.05). There were no significant alterations in serum CCL5 of IL-6 among the three groups after exposure.

### LC3II/I and total tau expressions

**Fig. 7:**
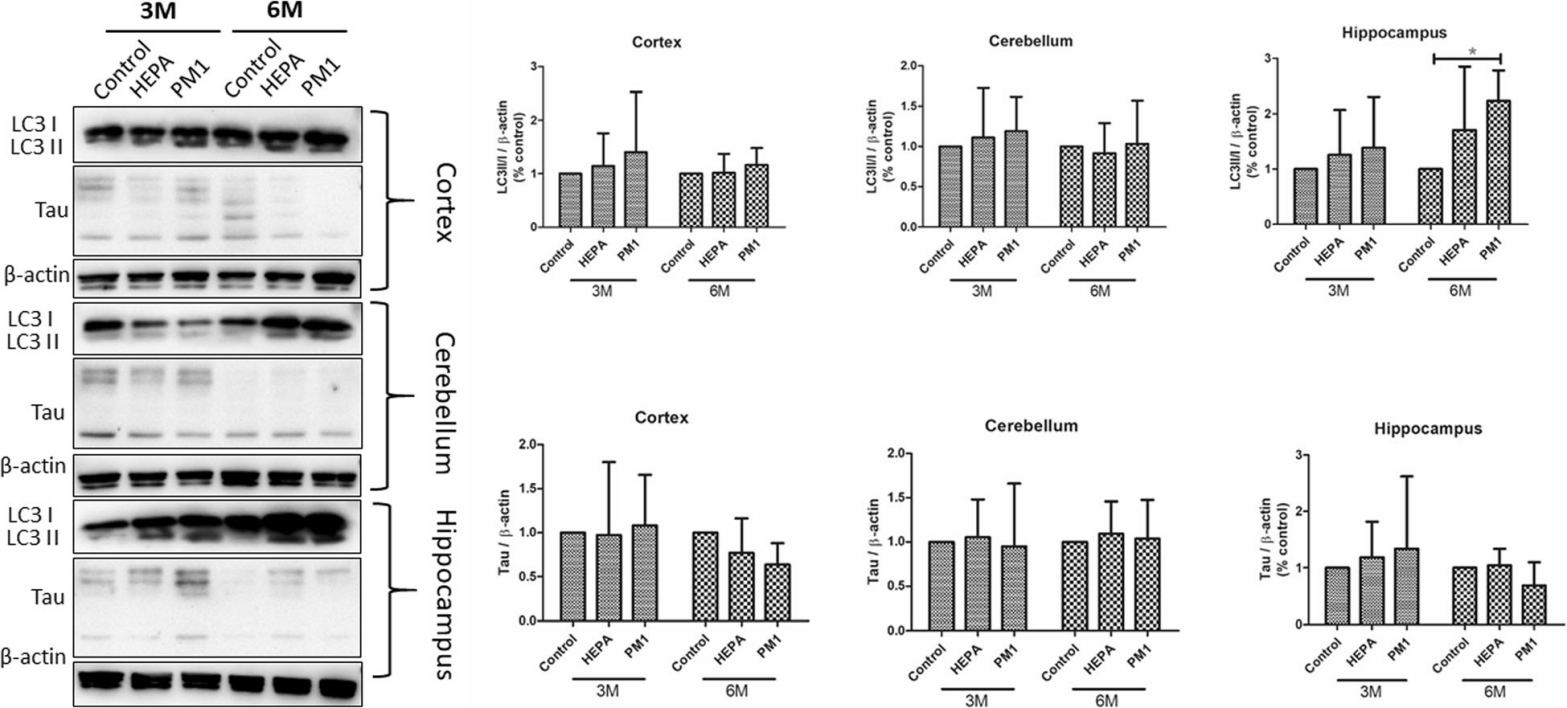
Expressions of light chain 3II and I (LC3II/I) and total tau in the cortex, cerebellum, and hippocampus of rats after 3 and 6 months of exposure to traffic-related air pollution in the control, high-efficiency particulate air (HEPA), and particulate matter with an aerodynamic diameter of < 1 μm (PM_1_) groups. LC3II/I expression in the hippocampus increased after 6 months of exposure to PM_1_. * *p* < 0.05

**Fig. 8:**
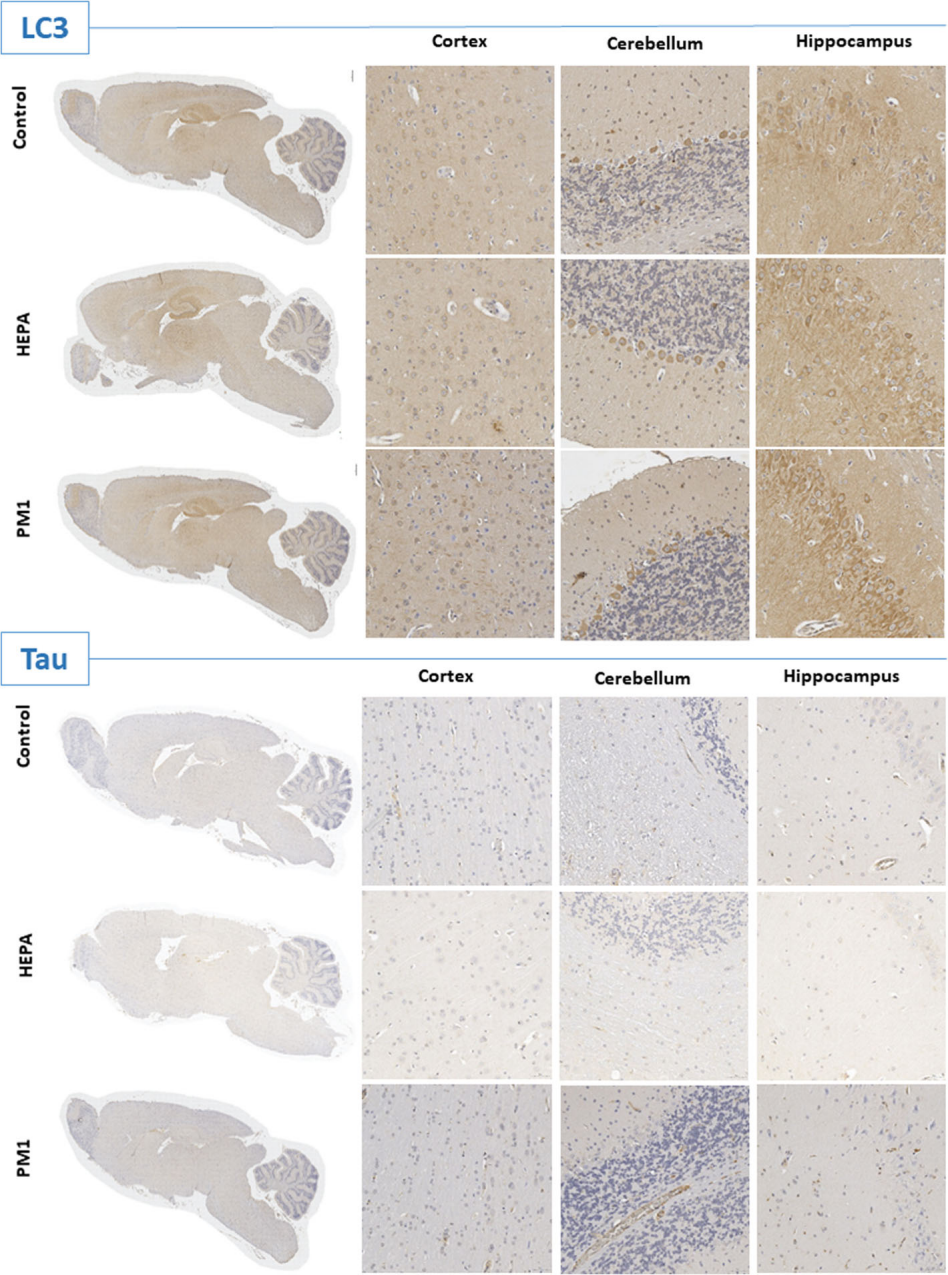
IHC images of light chain 3 (LC3) and total tau in the cortex, cerebellum, and hippocampus of rats after 6 months of exposure to traffic-related air pollution in the control, high-efficiency particulate air (HEPA), and particulate matter with an aerodynamic diameter of < 1 μm (PM_1_) groups. LC3 was more strongly positive in the hippocampus after 6 months of PM_1_ exposure. Scar bar is 50 μm

Expressions of LC3II/I and total tau proteins in the cortex, cerebellum, and hippocampus of rats in the control, HEPA, and PM_1_ groups after 3 and 6 months of exposure are shown in Fig. 7. We observed that the LC3II/I ratio was significantly higher after 6 months of exposure to PM_1_ compared to the control (*p* < 0.05). This observation was also confirmed by IHC images. We observed that LC3 expression was significant higher in the hippocampus after PM_1_ exposure (6 months; Fig. 8). However, we did not observe a significant difference in the LC3II/I ratio in the cortex or cerebellum among the three groups after exposure, as confirmed by the IHC analysis. There were no significant alterations in tau in the cortex, cerebellum, or hippocampus after 3 and 6 months of exposure. We also did not observe any changes in tau expression by the IHC analysis.

### Histology

**Fig. 9:**
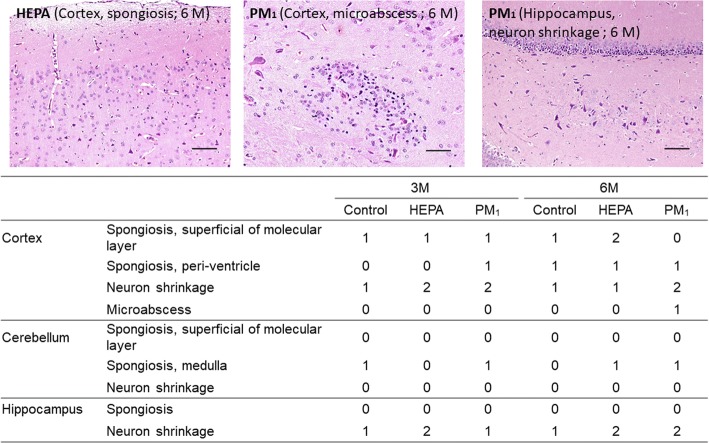
H&E staining and scoring of the cortex, cerebellum, and hippocampus after 3 and 6 months of exposure to traffic-related air pollution in the control, high-efficiency particulate air (HEPA), and particulate matter with an aerodynamic diameter of < 1 μm (PM_1_) groups. Spongiosis and neuronal shrinkage were observed after 3 and 6 months of exposure to PM_1_ (× 200). Microabscesses were identified in the cortex after 6 months of PM1 exposure (× 200). 0: No significant lesions. The extent of the lesions was graded from 1 to 5 depending on the severity: 1 = minimal (< 1%); 2 = slight, mild (1%~ 25%); 3 = moderate (26%~ 50%); 4 = moderate/severe (51%~ 75%); 5 = severe/high (76%~ 100%)

Figure 9 shows histological changes in the cortex, cerebellum, and hippocampus of rats in the control, HEPA, and PM_1_ groups after 3 and 6 months of exposure. We observed that PM_1_ caused periventricular spongiosis in the cortex after 3 months of exposure. Neuronal shrinkage in the cortex was also observed after 3 months of HEPA and PM_1_ exposure. Increased spongiosis in the superficial molecular layer of the cortex was observed after 6 months of HEPA exposure (as shown in Fig. 9). We also observed neuronal shrinkage in the cortex after 6 months of exposure to PM_1_. Notably, microabscesses were identified in the cortex after 6 months of PM_1_ exposure (as shown in Fig. 9). Spongiosis in the medulla was observed in the HEPA and PM_1_ groups after 6 months of exposure. Hippocampal neuronal shrinkage was observed after 6 months of exposure to HEPA and PM_1_ (as shown in Fig. 9).

## Discussion

Epidemiological reports indicated that exposure to air pollution is associated with neuropathologies that can instigate neurological disease. However, the pathophysiology of the brain that occurs due to particulate air pollution remains unclear. In the present study, the brain pathophysiology and neurotoxicity were investigated in adult rats after chronic pulmonary exposure to traffic-related PM_1_. Six major findings are reported in the present study: (1) the brain volume of rats increased after chronic exposure to PM_1_; (2) a deficiency in short-term memory occurred after sub-chronic exposure to PM_1_; (3) brain oxidative stress and hippocampal inflammation were caused by sub-chronic exposure to PM_1_; (4) systemic CCL11 and IL-4 increased after PM_1_ exposure; (5) autophagic activation by chronic PM_1_ exposure was observed in the hippocampus; and (6) spongiosis, neuronal shrinkage, and microabscesses were observed in the brain after PM_1_ exposure.

Traffic-related particulate air pollution has been linked to increased risks of neurological disorders [18]. To study the effects of traffic-related particulate air pollution on the brain, a traffic-dominated urban region in New Taipei City (Taiwan) was selected for the experimental site, where there are lanes with heavy vehicle frequency and a nearby highway and expressway. The temperature, RH, NOx, SO_2_, and O_3_ during the study periods were referenced from the nearby traffic-related Yonghe air quality monitoring stations (Additional file 2: Table S1), which are operated by the Taiwan Environmental Protection Administration (http://taqm.epa.gov.tw/taqm/tw/) [19]. The temperature was 20 ± 4 (min-max: 12~ 29) °C and RH was 72% ± 9% (min-max: 47%~ 92%). The gaseous pollutants were 32.9 ± 16.4 (min-max: 8.4~ 86.6) ppb for NOx, 2.5 ± 1.0 (min-max: 0.2~ 5.0) ppb for SO_2_, and 29.7 ± 11.0 (min-max: 6.7~ 58.2) ppb for O_3_ during the study period. All three gaseous pollutants were lower than WHO air quality guidelines [20] during the study. Notably, for particulate air pollution, the mean geometric mean diameter (GMD) was 55.8 nm during the study period (Fig. 2), which suggests that most of the particles the rats were exposed to were dominated by ultrafine-sized fractions. The PM_1_ mass concentration was 16.3 μg/m^3^, which is similar to our previous observation in Taipei City (Taiwan) [21, 22]. PM_1_ levels were relatively lower than the WHO PM_2.5_ guideline of 25 μg/m^3^ for the 24-h average [20]. However, there was 18 days of PM_1_ mass concentrations higher than the WHO 24-h mean PM_2.5_ guideline, and more than 116 days of PM_1_ mass concentrations higher than the WHO annual mean PM_2.5_ guideline. The rats were continually exposed to traffic-related PM_1_, the LDSA of which was 55.1 μm^2^/cm^3^ during the study period. The LDSA level was higher than measured levels (37 μm^2^/cm^3^) in Barcelona, Spain [23]. This result indicates that the particle size suspended in the ambient air in New Taipei City was smaller (a higher surface area of particles) than particles measured in Barcelona. The BC concentration (1,899 ng/m^3^) and PNC (11,257 #/m^3^) determined in the present study were higher than our previous measurements (1,229 ng/m^3^ for BC and 6,065 #/m^3^ for PNC) [21]. Based on measurements of gaseous and particulate air pollution, the rats were exposed to relatively lower traffic-dominated air pollution during the study period. Therefore, this exposure condition of air pollution provides a good platform to study effects of chronic exposure to air pollution on the brain.

To study the pathophysiology of the brain caused by 3 and 6 months of exposure to particulate air pollution, a clean air control group (control), HEPA group (for gaseous effects), and PM_1_ group were evaluated. First, a structural change in the brain by PM_1_ in rats was observed by the MRI analysis (Fig. 3). Surprisingly, for the first time, we observed that the whole brain volume in 3-month PM_1_-exposed rats was significantly larger than that of control rats. This finding suggests that sub-chronic exposure to PM_1_ enlarged the whole brain volume of rats. Furthermore, the whole brain and hippocampal volumes of rats were enlarged by 6 months of exposure to gaseous pollution and PM_1_. However, our in vivo observations were inconsistent with previous epidemiological associations. For example, Power and colleagues (2018) observed that long-term exposure to PM_2.5_ was associated with smaller deep-gray volume of the brain in the Atherosclerosis Risk in Communities study. Long-term exposure to PM_2.5_ was associated with increasing loss of both gray matter and white matter of brains of older women [24], but that was not observed for hippocampal volumes [25]. Wilker and colleagues (2015) observed that air pollution was associated with insidious effects on structural brain aging even in dementia- and stroke-free persons. Our observations in rats may have been due to the different statuses of disease progression in the brain due to air pollution. The BBB plays an important role in preventing entry of neurotoxic plasma-derived components, cells, and pathogens into the brain. A breakdown of the BBB may result in alterations in the brain’s structures, such as cerebral edema and atrophy. A recent study found that 3-month-old C57BL/6 mice exposed to 100 μg/m^3^ of vehicle emissions for 30 days exhibited increased BBB permeability and impaired BBB integrity [26]. Therefore, it is reasonable to hypothesize that chronic exposure to air pollution causes early brain pathologies, such as cerebral edema as observed in our study. Brain edema is defined as an increase in the brain volume as a result of abnormal accumulation of fluid within the cerebral parenchyma, which can be a fatal pathological state of the brain [27]. The abnormal accumulation of fluid due to an increase of the BBB permeability and integrity impairment can cause an increase in the brain volume and elevation of intracranial pressure because of the enclosed rigid skull. Generally, cerebral edema is mainly classified into vasogenic and cytotoxic edema [28, 29]. Vasogenic edema is characterized by extravasation and extracellular accumulation of fluid in the cerebral parenchyma caused by BBB injury, whereas cytotoxic edema is characterized by intracellular accumulation of fluid and Na^+^ resulting in cell swelling. In contrast, cerebral atrophy is characterized by loss of neurons, shrinkage of neuronal cell bodies, or reductions in the number and extent of dendrites [30], leading to a decrease in the brain volume. Based on our MRI observations, 6 months of exposure to air pollution induced early-phase cerebral edema. Also, the occurrence of edema was followed by cerebral atrophy in the late stage as reported by epidemiological evidence. A neuroimaging study in individuals with mild cognitive impairment and early Alzheimer’s disease (AD) showed BBB breakdown in the hippocampus [31] and several grey and white matter regions [32, 33], respectively. Alterations in the structural integrity of the brain, including cerebral edema and atrophy, may be hallmarks of pathophysiological changes for particulate air pollution-induced neurological disorders; however, further studies are required to examine the possible mechanisms.

Neuroimaging observations in our study showed that the brain and hippocampal volumes were enlarged by exposure to traffic-related air pollution. Clinical complications of cerebral edema include behavioral and cognitive changes, memory loss, mental dysfunction, etc. [34]. Therefore, we next observed behavioral changes in rats by the NOR task (for short-term memory) and rotarod performance test (for motor function) (Fig. 4). We found that a short-term memory deficiency was caused by 3 months of exposure to PM_1_ in rats. Consistently, associations between cognitive dysfunction and air pollution were previously reported. For example, exposure to PM was related to a decrease in cognition function [1] and faster cognitive declines [2]. Exposure to PM_2.5_ and NO_2_ was associated with decreased cognitive function [35]. Similarly, impairments in spatial learning and memory were also observed in mice after exposure to PM_2.5_ [36]. A previous report showed that mild cognitive dysfunction is a prodromal syndrome of neurodegenerative dementia without significant dysfunction in activities of daily living [37]. Their observations support our finding of insignificant alteration in motor function after exposure to air pollution in rats. In the present study, the behavioral observations support the MRI results that PM caused neurotoxicity. However, the underlying mechanisms and pathophysiology of air pollution-induced cognitive dysfunction remain unclear.

Next, quantitative electroencephalography (qEEG) was carried out in rats after exposure (Fig. 5). qEEG measures are considered a reliable technique to examine neurodegenerative diseases, such as PD and AD, at the beginning of the dementing process, and can also be correlated with the extent of cognitive decline [38–40]. The presence of PD was correlated with an increase in theta power in the left temporal region and a decreasing median frequency [38]. Also, a human study was conduced to study alterations of the median power frequency and specific frequency bands of the qEEG after exposure to dilute diesel exhaust at 300 μg/m^3^ [41]. The authors observed a significant increase in the median power frequency (fast wave activity of beta2) in the frontal cortex with 30 min of exposure. Therefore, we expected that the results of qEEG would confirm the behavioral observations in the present study. However, there was no significant difference in the relative or absolutely power of the frequency band in rats. EEG signals are produced by nonlinear coupling interactions between neuronal cells. Neuronal death and a deficiency of neurotransmitters such as acetylcholine and/or loss of connectivity of local neuronal networks are thought to be associated with a decrease in the dynamic complexity of the EEG in neurodegenerative disease [42]. But, we did not observe significant differences in the MSE among the control, HEPA, and PM_1_ groups in the present study. The qEEG measurement reflects functional connections between different brain sections beneath the electrodes. The insignificant observation could have been due to a difference between the pathological location and the connected electrodes in the brain. Also, 6 months of exposure to air pollution will not cause fatal injuries to neuronal cells in the brain.

Neuroinflammation, a non-specific protective response, is recognized as an essential response to exposure to particulate air pollution [4, 5]. Chronic inflammation is observed in aged brains [43], and there are further increases in brain inflammation in neurodegenerative disorders. In the present study, we observed structural changes in the brain and behavioral alterations in rats after pulmonary exposure to traffic-related air pollution. It is important to further explore the bioreactivity of PM_1_ on various brain sections, including the cortex, cerebellum, and hippocampus. We observed that PM_1_ produced oxidative stress in the cortex, cerebellum, and hippocampus after 3 months of exposure in rats (Figure 6). Also, we found that hippocampal inflammation was caused by 3 months of exposure to PM_1_. Oxidative stress is considered an important mechanism in regulating inflammatory responses [44, 45], which was observed in our study. Oxidative stress is commonly observed in neurodegenerative diseases, which is recognized to be neurodegenerative pathogenesis, and it is conceivable that the effects of PM exposure could induce a decrease in cognitive function [46]. Acute exposure to 250~ 300 μg/m^3^ diesel exhaust particle for 6 h was found to induce oxidative stress, microglia activation, neuro-inflammation, and neurogenesis impairment in various brain regions, such as the hippocampal subgranular zone and subventricular zone [47]. Long-term exposure to PM_2.5_ elevated hippocampal inflammatory cytokines in mice [36]. Campbell and colleagues (2005) observed that asthmatic mice exposed to concentrated ambient particles exhibited increased inflammatory cytokines, such as IL-1α and tumor necrosis factor (TNF)-α in brain tissues. Guerra and colleagues (2013) further showed that exposure to different-sized fractions of ambient particles caused distinct physiological changes, inflammation, oxidative stress, and unfolded protein responses in the CNS, particularly in the striatum. Although in vivo studies indicated that particulate air pollution causes neuroinflammation, possible lung-to-brain pathways of exposure to air pollution remain unclear. It was noted that ultrafine PM is capable of being translocated into the brain via the olfactory nerves [13]. Also, inhaled PM is able to damage the nasal epithelium [14]. To examine the effects of PM_1_ on the olfactory bulb, oxidative stress, inflammation, and cytotoxicity were determined in rats after 3 and 6 months of PM_1_ exposure. However, we did not observe significant alterations in 8-isoprostane, IL-6, or lactate dehydrogenase (LDH) levels with PM_1_ exposure. A previous study showed that exposure to ambient PM induced olfactory bulb inflammatory gene expressions in C57BL/6 male mice [48]. The difference from the current study could have been due to different expression levels between proteins and genes. Notably, one study observed that exposure to traffic-related air pollution altered the brain’s microvascular integrity in a high-fat diet animal model [26]. The authors suggested that particulate air pollution could cause BBB impairment, leading to local inflammation due to particles accumulating in the brain. If such BBB impairment is an important pathway of regulating air pollution-induced neurotoxicity, an increase in the permeability of the brain and systemic-to-local (brain) inflammation could explain the MRI observations of increased brain volumes. Additionally, we observed that CCL11 and IL-4 had increased after 3 and 6 months of exposure to PM_1_ in rats. CCL11 was shown to pass the BBB and was identified as a crucial mediator of decreased neurogenesis and increased cognitive impairment in mice [49]. Mice lacking IL-4 demonstrated cognitive impairment in a spatial learning task [50]. Together, alterations in CCL11 and IL-4 could support the behavioral observations in the present study. However, additional mechanistic investigations should be conducted in the future.

Deposition of insoluble proteins in cells of the neuromuscular system is related to the development of neurological diseases. Clinically, intraneuronal accumulation of tau proteins is considered an important hallmark of the development of AD [51]. Tau overexpression in neuroblastoma cells can lead to tau aggregation and the appearance of smaller proteolytic fragments [52]. In the present study, we did not observe significant alterations in tau expression in the cortex, cerebellum, or hippocampus after 3 or 6 months of exposure to air pollution in rats (Figs. 7 and 8). Our results suggest that exposure of air pollution in rats did not produce significant development of neurodegenerative disease at the current stages. A previous study showed that phospho-tau (Ser199) increased after exposure to diesel exhaust at 992 and 311 μg/m^3^ [53]. Notably, we observed autophagy activation in the hippocampus after 6 months of exposure to PM_1_ in rats. Autophagy is one of the degradative mechanisms, which is able to remove tau aggregates [52]. Therefore, autophagy is considered an essential function for maintaining the brain’s health. Autophagy dysfunction and loss of basal autophagy may lead to neurodegeneration, whereas activation of autophagy can remove aggregated oxidized/diseased proteins such as tau protein aggregates [54]. In the present study, we suspect that activation of autophagy may have played a critical role in maintaining the brain’s health after exposure to air pollution.

Due to the rigidity of the skull, cerebral edema can lead to higher intracranial pressure, which subsequently decreases cerebral perfusion, ultimately leading to pathological changes in the brain [55]. In the present study, spongiosis and/or neuronal shrinkage were commonly observed in the cortex, cerebellum, and hippocampus of rats exposed to air pollution (Fig. 9). Fonken and colleagues [36] observed that exposure to PM_2.5_ for 10 months caused changes in the hippocampal neuronal morphology, including apical dendritic spine density and dendritic branching, in mice. We further showed that microabscesses occurred in the cortex after 6 months of exposure to PM_1_. Brain abscesses, defined as focal infections within the brain parenchyma, are caused by inflammation and the collection of infected material within brain tissues [56]. Therefore, the cortical microabscesses observed after 6 months of exposure to PM_1_ may have resulted from particles crossing the BBB into the brain. However, more-direct evidence is required to examine the possible pathways of PM-related neurotoxicity.

**Fig. 10:**
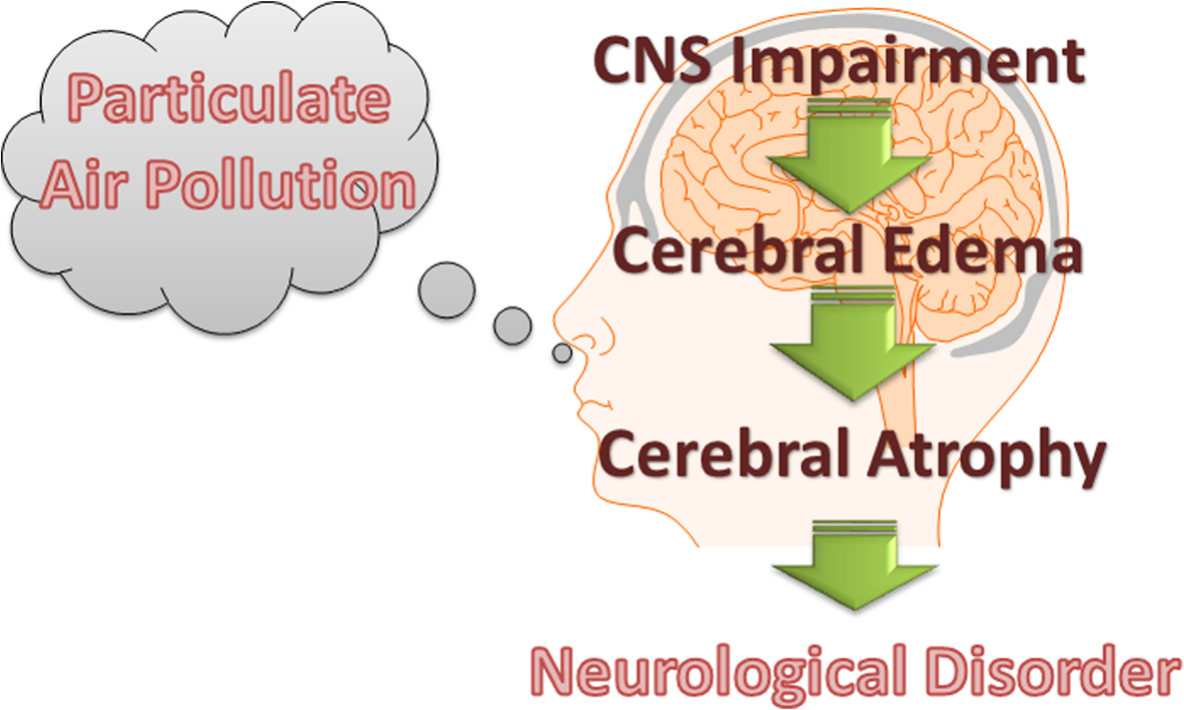
Illustration of the hypothetical pathways of particulate air pollution involved in the development of neurological disorders. Inhaled particulate air pollution is able to induce oxidative stress and inflammation in the brain, leading to central nervous system (CNS) impairment. The extracellular accumulation of fluid due to increased permeability of the blood-brain barrier (BBB), results in formation of cerebral edema. The air pollution-induced edematous brain is repaired by protective mechanisms, leading to cerebral atrophy. Continuous exposure to particulate air pollution leads to the development of neurological disorders

Taken together, we hypothesized a theoretical model for the impacts of particulate air pollution on the brain as shown in Fig. 10. Inhaled particulate air pollution is able to induce oxidative stress and inflammation in the brain, leading to CNS impairment. The extracellular accumulation of fluid due to the increased permeability of the BBB results in the formation of cerebral edema. The air pollution-induced edematous brain is repaired by protective mechanisms, resulting in cerebral atrophy. Although our results support the hypothetical model of PM neurotoxicity, cerebral atrophy was not observed in the present study (due to the stage of pathological progression). Also, the pathways of PM from the lungs to the brain remain unclear. We found that gaseous pollution caused neurotoxicity after 6 months of exposure, including increase in whole and hippocampus volumes and systemic CCL5 levels in rats. The gaseous effect on brain is poorly unknown. Based on our findings, we hypothesize that some types of gaseous pollution is a source of free readcals that could cause systemic oxidative stress and inflammation, leading to brain impariement. However, the contributions of different gaseous pollutants were not investigated in the present study. Effects of metals and organics on neurotoxicity should be addressed in future work.

## Conclusions

In conclusion, chronic exposure to air pollution caused brain impairment, leading to cerebral edema, behavioral changes, oxidative stress, inflammation, autophagy activation, and histological changes in the rat brain. Cerebral edema could be an early stage of progression for the development of neurological diseases caused by air pollution. Our findings provide further evidence that long-term exposure to particulate air pollution increases the risks of neurological disorders.

## Materials

### Animals

Animal experiments were performed in compliance with the animal and ethics review committee of the Laboratory Animal Center at Taipei Medical University (Taipei, Taiwan). Male 6-month-old SD rats obtained from the National Laboratory Animal Center (Taipei, Taiwan) were maintained at a constant temperature of 22 ± 2 °C and a relative humidity (RH) of 55% ± 10%. Rats were housed in plastic cages with a 12:12-h light: dark cycle throughout the study. Lab Diet 5001 (PMI Nutrition International, USA) and water were provided ad libitum during the study.

### Whole-body exposure to traffic-related PM_1_

As shown in Fig. 1a, rats were randomly divided into three groups: (1) a clean air control group (housed in the Specific Pathogen Free I (SPF I) level of Laboratory Animal Center, Taipei City), (2) a high-efficiency particulate air (HEPA)-filtered air control group (exposed to gaseous pollution only), and (3) a PM_1_ group (exposed to particulate and gaseous pollution). Because of ventilation of the animal centre was introduced from the outdoor ambient air with HEPA filtration, the clean air control group may be still exposed under the urban background levels of air pollution. The whole-body exposure system used to radent for PM_1_ exposure was previously reported [22]. The whole-body exposure system was located in a traffic-dominated urban region (New Taipei City, Taiwan), which is near a highway and expressway. Briefly, ambient air was continuously sampled by an omnidirectional PM inlet located on the roof of a station and then introduced into whole-body exposure system as shown in Fig. 1b. A stream was sampled from an empty whole-body exposure cage (without rats) for characterization of PM’s physical features. The whole-body exposure system had the higher penetration rate of PM (outdoor PM to system PM) with the particle size less than 1 μm (Additional file 2: Figure S2). The data suggested that PM_1_ was the main size fraction for the whole-body exposure in this system. Mass concentrations were monitored using a tapered element oscillating microbalance (TEOM, Thermo Scientific 1400a). Additionally, a scanning mobility particle sizer (SMPS, TSI 3936), an aerodynamic particle sizer (APS; TSI 3321), and a nanoparticle surface area monitor (NSAM; TSI 3550) were respectively used to monitor the submicron particle size distribution (PSD), supermicron PSD, and LDSA concentrations. An aethalometer (Magee Scientific AE33, Berkeley, CA, USA) was simultaneously employed to measure the BC mass concentration. The details of these instrutemnts are provided in Additional file 2: Table S2. The onsite exposure experiment was conducted for 3 and 6 months.

### Experimental design

The experimental design is shown in Fig. 1c. Rats in the HEPA (*n* = 18) and PM_1_ groups (*n* = 18) were directly exposed to traffic-related air pollution for 3 and 6 months between November 2016 and May 2017. Simultaneously, rats in the clean air control group (*n* = 18) were housed in the Laboratory Animal Center for the same study period (3 and 6 months). After exposure for 3 and 6 months, nine rats in each group were randomly selected for an MRI analysis. Next, six rats in each group, after the MRI, were randomly selected for behavioral observations, including a novel object recognition (NOR) task and rotarod performance task. Electroencephalographic (EEG) implementation and monitoring were conducted in the remaining three rats from each group. Finally, an animal necropsy was performed, and serum and tissues were collected as described previously [57].

### MRI

MRI experiments were performed on a custom-made 3 T MRI system. A gradient coil insert with an inner diameter of 115 mm and a maximum gradient strength of 670 mT/m (BFG 200–115, Resonance Research, Inc., USA) was installed and a home-made single-loop surface RF coil with a diameter of 4 cm was used for RF transmission. MRI sequences were executed by a 3 T spectrometer (MR Solutions, UK). For rat brain imaging, T2-weighted images (T2WIs) were acquired using a fast spin echo sequence with 10 coronal slices, a slice thickness of 2 mm without a gap, a repetition time (TR) of 4000 ms, an echo time (TE) of 63 ms, an echo train length (ETL) of 8, a field of view (FOV) of 40 × 40 mm, and a matrix size of 256 × 256, yielding an in-plane resolution of 156 × 156 μm, with 16 averages. All animals were fixed with a home-made magnetic resonance-compatible animal stereotaxic frame, made using a 3D printer (KINGSSEL3070+, MASTECH MACHINE CO., LTD., Taiwan), and they were anesthetized using an isoflurane vaporizer with the concentration adjusted to maintain 40~ 60 breaths/min of the breathing rate during the MRI scan. The total experiment time was around 1 h for each animal.

### MRI data analysis

For the MRI volumetric analysis, the bilateral hippocampus and whole brain areas were manually selected using MRI analytical software, MRIcro (https://www.mccauslandcenter.sc.edu/crnl/). The region of interest (ROI) of the hippocampus was determined according to the rat brain atlas [58]. The voxel numbers of each ROI were automatically calculated using MRIcro. The volume calculation was based on the counted voxel numbers multiplied to the voxel size of the acquired image (0.156 × 0.156 × 2 mm).

### NOR task

The NOR task was reported to be a useful approach for studying short-term memory, which is influenced by both hippocampal and cortical lesions. The procedure of NOR task was previously reported [59]. Briefly, the NOR sample and test sessions occurred in context A, an open field arena measuring 60 × 60 cm, with 40-cm-high black translucent walls and a white floor with black gridlines spaced 15 cm apart. Context A was 360° encompassed by a 180-cm-high black curtain in order to block distal sensory information; object locations inside the arena also remained constant between sessions. Over 3 days, each subject was transported in its home cage and allowed to explore context A for 5 min, after which it was returned to the vivarium. During the sample and test sessions, objects were taped to the floor to ensure that they could not be moved by the animal. The objects were a transparent green water bottle made of non-porous plastic (22 × 9 cm) and a semi-opaque, brown glass bottle (22 × 7 cm). The two objects were located 15 cm from the nearest arena wall and separated by 30 cm. For the sample session, each rat was placed near the center of the open field, facing away from the two identical objects (counter-balanced) and allowed to explore for 5 min. Rats were returned to the same context 20 or 240 min later. Using the same spatial locations within the arena, one familiar object (sample objective) and one novel object were presented, counter-balanced across the left-right position. The subject was again placed near the center of the open field, facing away from the objects and allowed to explore for 3 min. The recognition index, the proportion of time spent with the novel object/location compared to the total exploration time of both objects, was used to measure recognition memory. A recognition index significantly above 0.50 demonstrates a novelty preference and thus recognition memory [60].

### Rotarod performance test

The rotarod performance test was carried out using the Rotarod system (Rotamex-5; Columbus Inst; Columbus, OH, USA) to evaluate motor function. Each rat was trained three times/day for 3 consecutive days before the real test. The training speed was from 4 to 20 revolutions/min (rpm) at a rate of 0.5 revolutions/s (rps). After completing the training, a rat was placed onto a rotating rod at a constant speed of 40 rpm over a period of 5 min essentially, acceleration Increments of 1.2 rpm per ten second. The duration time of staying on the rotating rod was recorded. Each rat was given three trials at 30-min intervals, and the average time of the three trials was recorded as the final result.

### EEG implementation and monitoring

Stereotactic surgery was conducted under light anesthesia induced by 2% isoflurane vapor (2 ml/min) using a rodent anesthesia machine (Northern Vaporiser; Skipton, UK). EEG electrodes were secured to the skull using stainless steel screws, which were placed epidurally in the frontal cortex, parietal area (somatosensory cortex), and occipital cortex [61]. Electrodes were fixed to the skull with methyl methacrylate monomer together with two additional anchoring screws. Simultaneously, electromyography (EMG) was implemented and connected to a muscle. After surgery, rats were individually housed for 7 days in order to prevent damage to the electrode connectors. After recovery, EEG signals were continuously recorded for 24 h in freely moving rats, and the output was fed into a multi-channel amplifier (MP35, Biopac System, Goleta, CA, USA). The sampling frequency of raw EEG data was 200 Hz, and the digital resolution was 12 bits. A notch filter of 50/60 Hz was applied.

### Power spectrum analysis of EEG

EEG data were mainly processed using MATLAB software (MATLAB, R2007a; MathWorks, Natick, MA, USA) to conduct further computations. First, raw EEG data were visually checked to exclude epochs with significant artifacts. Fast Fourier transformation of the EEG data was computed to obtain the power spectrum of the EEG signals. The absolute power and relative power of four frequency bands were calculated, including the delta (0.5~ 2 Hz), theta (2~ 8 Hz), alpha (8~ 12 Hz), and beta (12~ 30 Hz) bands.

### Multiscale entropy (MSE) analysis

MSE was determined based on an analysis of sample entropy of EEG signals at different time scales. Signals at differential time scales were computed through a coarse-graining procedure of the original data. The coarse-graining procedure was conducted by dividing the original signal into non-overlapping segments of equal length corresponding to the scalar factor. The average of signals in each window was then calculated to yield a new time series. The sample entropy, calculated as the self-similarity of the signal based on the conditioned probability, was then calculated for each time series based on different scalar factors. We utilized the C -ode program developed by Goldberger et al. to calculate the MSE of the EEG signals [62].

Given a time series denoting the measured EEG:

1 $$ \mathrm{x}\left(\mathrm{n}\right)=\left(\times 1,\times 2,\times 3,\dots .,\mathrm{xn}\right); $$

the coarse-grain series was obtained by averaging variables by applying non-overlapping windows.

2 $$ \mathrm{X}\left(\mathrm{s}\right)=\left(\mathrm{mean}\;\left(\times 1,\times 2,\times 3,\dots .,\mathrm{x}\mathrm{s}\right),\kern0.37em \mathrm{mean}\;\left(\mathrm{xs}+1,\mathrm{x}\mathrm{s}+2,\mathrm{x}\mathrm{s}+3,\dots .,\mathrm{x}2\mathrm{s}\right),\dots .\right); $$

the MSE for each time scalar factor was computed according to the equation:

3 $$ \mathrm{MSE}\ \left(\mathrm{s}\right)=\mathrm{SpEn}\;\left(\mathrm{X}\left(\mathrm{s}\right),\mathrm{m},\mathrm{r}\right); $$

where SpEn denotes the sample entropy, m denotes the vector of length, and r denotes the tolerance of similarity. In the present study, we selected m = 2 and *r* = 0.15 for MSE computations.

### Brain and olfactory bulb tissue preparations

Rats from each group (control, HEPA, and PM_1_) were transcardially perfused with 15 mL of phosphate-buffered saline (PBS) and immediately decapitated. The cortex, cerebellum, hippocampus, and olfactory bulb from each rat were collected, frozen in liquid nitrogen, and stored at − 80 °C. Tissues were homogenized in Cell lysis MT (Sigma, St. Louis, MO, USA) with a Complete™ protease inhibitor (Roche Diagnostics, Basel, Switzerland), according to the manufacturers’ instructions. All preparation were conduced on ice, and centrifuged for 30 min at 12,000 x*g* and 4 °C. The supernatant was collected, and the total protein concentration was measured using a BCA Protein Assay Kit.

### Enzyme-linked immunosorbent assay (ELISA)

8-Isoprostane (Cayman, USA), IL-6 (R&D System, Minneapolis, MN, USA) and Lactate dehydrogenase (LDH) Cytotoxicity Assay Kit (Thermo Scientific, Waltham, MA, USA) were determined using ELISA kits in accordance with the manufacturer’s instructions. All data are presented after adjusting for the total protein.

### Multiplex assay

Chemokine (C-C motif) ligand 5 (CCL5), CCL11, IL-4, and IL-6 in plasma were assayed with the AimPlex™ multiplex assay (St. Louis, MO, USA), according to the manufacturer’s instructions. Complexes of beads and the studied proteins labeled with phycoerythrin antibodies were determined using a BD LSRFortessa™ cell analyzer (NJ, USA).

### Western blot analysis

Details of the Western blot analyses were previously described [63]. Lysed tissue samples were electrophoresed through sodium dodecylsulfate polyacrylamide gel electrophoresis (SDS-PAGE) and transferred onto polyvinylidene difluoride (PVDF) membranes (Millipore, Darmstadt, Germany). Primary antibodies for light chain 3 (LC3; 1:1000), tau (1:1000), and β-catenin (1:1000) were obtained from Cell Signaling (Danvers, MA, US). Anti-rabbit (1:2000) and anti-mouse (1:2000) horseradish peroxidase (HRP)-conjugated secondary antibodies were obtained from Chemicon International (MA, USA) and Merck Millipore (MA, USA), respectively. PBST containing 5% skim milk was used to block blots, followed by probing with primary antibodies overnight at 4 °C. Samples were then incubated with an HRP-labeled secondary antibody at room temperature. Immunoreactivity was observed through enhanced chemiluminescence (ECL). Images were taken with the BioSpectrum Imaging System (UVP, Upland, CA, USA). Quantitative data were obtained using Image-Pro vers. 4 (Media Cybernetics, Inc., MD, USA) for Windorws. All data were adjusted to the control (multiples of change of the control).

### Immunohistochemistry (IHC) of brain tissues

Tissues were fixed in 2% paraformaldehyde and were permeabilized with 0.1% Triton X-100 in 0.01 M PBS (pH 7.4; containing 0.2% bovine serum albumin). Incubation with polyclonal antibodies against LC3 (1:1000) and tau (1:1000), obtained from GeneTex (Irvine, CA, USA), in PBS and containing 3% normal goat serum, was performed, whereas incubation with PBS served as a negative control. An anti-rabbit immunoglobulin G (IgG) FITC-conjugated secondary antibody (Jackson ImmunoResearch, PA, USA; 1:500 dilution) was incubated with cells, followed by staining with 4′,6-diamidino-2-phenylindole (DAPI). Microphotographs were acquired using an AxioCam MRc digital video camera and the Zeiss AxioVision software (Carl Zeiss, NY, USA).

### Histology

Brains were excised and washed with ice-cold PBS followed by fixation with 10% neutral buffered formalin, embedded in paraffin, sectioned, and stained with hematoxylin and eosin (H&E). Histological examinations were conducted under light microscopy by a histopathologist in a blinded manner.

### Statistical analysis

Data are expressed as the mean ± standard deviation (SD). For comparisons among multiple values, a one-way analysis of variance (ANOVA) with Tukey’s post-hoc test was used. Statistical analyses were performed using GraphPad vers. 5 for Windows. The level of significance was set to *p* < 0.05.

## Additional files

Additional file 1: **Figure S1.** Alteration in body weight during the 6-months exposure of PM_1_. (TIF 146 kb)

Additional file 2: **Table S1.** Meteorological and gaseous data measured by the traffic-related EPA Yonghe air quality monitoring stations during the study period. **Table S2**. Instruments used to characterize the exposure conditions for rats. **Figure S2**. Characterization of particle size and penetration distribution (between outdoor and whole-body exposure system) determined using a scanning mobility particle sizer (SMPS, TSI 3936; upper size limit: 710 nm). (a) The exposure cages (yellow marked: 1–1, 1–3, 1–5, 2–3, 3–1, 3–3 and 3–5) were measured for size-penetration distribution. (b) The individual cage for animal exposure showed a consistent size-penetration distribution. The geometric mean diameter (GMD) was 50 nm. (DOCX 152 kb)
