# Supplementary figures and images for: Chronic pulmonary exposure to traffic-related fine particulate matter causes brain impairment in adult rats
Source: Part Fibre Toxicol. 2018 Nov 9;15:44. doi: 10.1186/s12989-018-0281-1 (PMC6234801; doi:10.1186/s12989-018-0281-1)

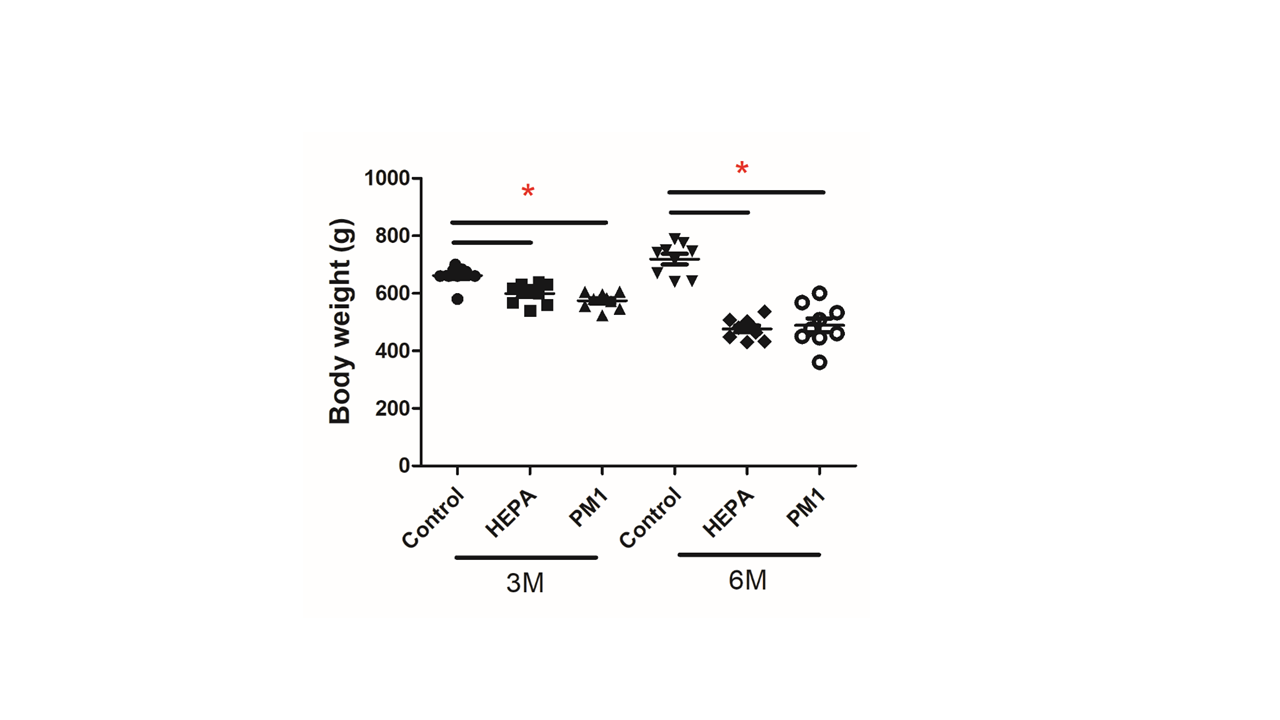

Supplement: Supplementary file 1 — Figure S1. Alteration in body weight during the 6-months exposure of PM1. (TIF 146 kb) [file 12989_2018_281_MOESM1_ESM.tif]
