# Supplementary material for: Chronic pulmonary exposure to traffic-related fine particulate matter causes brain impairment in adult rats
Source: Part Fibre Toxicol. 2018 Nov 9;15:44. doi: 10.1186/s12989-018-0281-1 (PMC6234801; doi:10.1186/s12989-018-0281-1)
Supplement: Supplementary file 2 — Table S1. Meteorological and gaseous data measured by the traffic-related EPA Yonghe air quality monitoring stations during the study period. Table S2. Instruments used to characterize the exposure conditions for rats. Figure S2. Characterization of particle size and penetration distribution (between outdoor and whole-body exposure system) determined using a scanning mobility particle sizer (SMPS, TSI 3936; upper size limit: 710 nm). (a) The exposure cages (yellow marked: 1–1, 1–3, 1–5, 2–3, 3–1, 3–3 and 3–5) were measured for size-penetration distribution. (b) The individual cage for animal exposure showed a consistent size-penetration distribution. The geometric mean diameter (GMD) was 50 nm. (DOCX 152 kb) [file 12989_2018_281_MOESM2_ESM.docx]

# Chronic pulmonary exposure to traffic-related fine particulate matter causes brain impairment in adult rats

Chi-Hsiang Shih^1^, Jen-Kun Chen^2^, Li-Wei Kuo^2^, Kuan-Hung Cho^2^, Ta-Chih Hsiao^3^, Zhe-Wei Lin^1^, Yi-Syuan Lin^1^, Jiunn-Horng Kang^4,5^, Yu-Chun Lo^6^, Kai-Jen Chuang^7,8^, Tsun-Jen Cheng^9^, Hsiao-Chi Chuang^1,7,10*^

^1^School of Respiratory Therapy, College of Medicine, Taipei Medical University, Taipei, Taiwan

^2^Institute of Biomedical Engineering & Nanomedicine, National Health Research Institutes, Miaoli, Taiwan

^3^Graduate Institute of Environmental Engineering, National Taiwan University, Taipei, Taiwan

^4^Department of Physical Medicine and Rehabilitation, Taipei Medical University Hospital, Taipei, Taiwan

^5^Department of Physical Medicine and Rehabilitation, School of Medicine, College of Medicine, Taipei Medical University, Taipei, Taiwan

^6^The Ph.D Program for Neural Regenerative Medicine, College of Medical Science and Technology, Taipei Medical University, Taipei, Taiwan

^7^School of Public Health, College of Public Health, Taipei Medical University, Taipei, Taiwan

^8^Department of Public Health, School of Medicine, College of Medicine, Taipei Medical University, Taipei, Taiwan

^9^Institute of Occupational Medicine and Industrial Hygiene, College of Public Health, National Taiwan University, Taipei, Taiwan

^10^Division of Pulmonary Medicine, Department of Internal Medicine, Shuang Ho Hospital, Taipei Medical University, New Taipei City, Taiwan

*** Corresponding Author**

*Hsiao-Chi Chuang, PhD*

Taiwan CardioPulmonary Research (T-CPR) Group, School of Respiratory Therapy, College of Medicine, Taipei Medical University, 250 Wuxing Street, Taipei 110, Taiwan.

Telephone: +886-2-27361661 ext. 3512. Fax: +886-2-27391143. E-mail: r92841005@ntu.edu.tw

**Table S1. Meteorological and gaseous data measured by the traffic-related EPA Yonghe air quality monitoring stations during the study period**

| Meteorological and gaseous data (unit) | Mean ± SD (Min~Max) |
| --- | --- |
| Temperature (°C) | 20 ± 4 (12~29) |
| Relative humidity (%) | 72 ± 9 (47~92) |
| NOx (ppb) | 32.9 ± 16.4 (8.4~86.6) |
| SO_2_ (ppb) | 2.5 ± 1.0 (0.2~5.0) |
| O_3_ (ppb) | 29.7 ± 11.0 (6.7~58.2) |

NOx: nitrogen oxides; SO_2_: sulfur dioxide; O_3_: ozone.

**Table S2. Instruments used to characterize the exposure conditions for rats**

| Instrument | Pollutant (unit) | Flow rate (lpm) | | Time resolution | |
| --- | --- | --- | --- | --- | --- |
| TEOM, Thermo Scientific 1400a | PM_1_ (µg/m^3^) | 3 | 5 min | |  |
| SMPS, TSI 3080 | PNC (#/cm^3^) | 0.6 | 5 min | |  |
| APS, TSI 3321 | PNC (#/cm^3^) | 5 | 5 min | |  |
| NSAM, TSI 3550 | LDSA (µm^2^/cm^3^) | 2.5 | 5 min | |  |
| Magee, AE-33 | BC (ng/m^3^) | 5 | 1 min | |  |
| AIO Weather, Climatronics | RH (%)  Temperature (^o^C) | ̶ | 1s | |  |

TEOM: tapered element oscillating microbalance; SMPS: scanning mobility particle sizer; APS: aerodynamic particle sizer; NSAM: nanoparticle surface area monitor; AE: Aethalometer; PM_1_: particulate matter less than 1 μm in aerodynamic diameter; PNC: particle number concentration; LDSA: lung deposition surface area; BC: black carbon; RH: relative humidity.


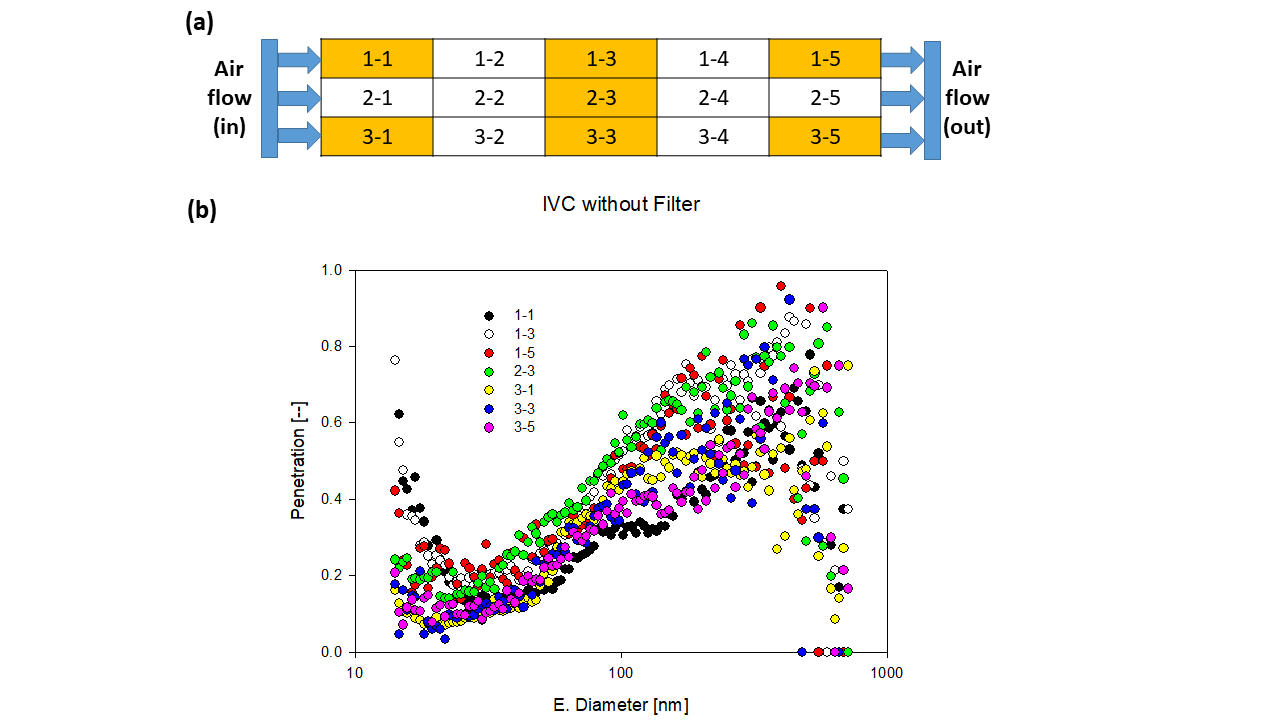


**Figure S1. Characterization of particle size and penetration distribution (between outdoor and whole-body exposure system) determined using a scanning mobility particle sizer (SMPS, TSI 3936; upper size limit: 710 nm). (a) The exposure cages (yellow marked: 1-1, 1-3, 1-5, 2-3, 3-1, 3-3 and 3-5) were measured for size-penetration distribution. (b) The individual cage for animal exposure showed a consistent size-penetration distribution. The geometric mean diameter (GMD) was 50 nm.**
